# Hepatocyte Differentiation from Human ES Cells using the Simple Embryoid Body Formation Method and the Staged-Additional Cocktail

**DOI:** 10.1100/tsw.2009.97

**Published:** 2009-09-01

**Authors:** Katsunori Sasaki, Hinako Ichikawa, Shunsuke Takei, Hee Sung No, Daihachiro Tomotsune, Yoshiya Kano, Tadayuki Yokoyama, Sakiko Sirasawa, Akimi Mogi, Susumu Yoshie, Shujiro Sasaki, Satoshi Yamada, Ken Matsumoto, Masahiro Mizuguchi, Fengming Yue, Yoshiki Tanaka

**Affiliations:** ^1^ Department of Histology and Embryology, Shinshu University, School of Medicine, 3-1-1 Asahi Matsumoto, Nagano 390-8621, Japan; ^2^ Bourbon Corporation, 44-2-14 Matsunami, Kashiwazaki City, Niigata 945-8611, Japan; ^3^ NOF Corporation, 5-10 Tokodai, Tsukuba, Ibaraki 300-2635, Japan; ^4^ Nissui Pharmaceutical Co., Ltd, 1075-2 Hokunanmoro, Yuki-shi, Ibaraki 307-0036, Japan

**Keywords:** hES cells, EB formation, hepatocyte differentiation, suspension culture, Lanford medium, microscopy

## Abstract

To induce hepatocytes from human embryonic stem (hES) cells easily and effectively, a simple suspension culture method that separates ES colonies with a scraper and transfers them into newly developed, nonadherent MPC (2-methacryloyloxyethyl phosphorylcholine) plates, and the staged-additional cocktail method, including growth factors, cytokines, and Lanford serum-free medium, were developed and evaluated mainly by morphological analysis. The formed embryoid bodies (EBs) showed compact cellular agglomeration until day 4 and later formed coeloms in their interior. RT-PCR (reverse transcriptase-polymerase chain reaction) analysis showed that they are gene markers of the three germ layers. Mesenchymal cells with rough endoplasmic reticulum (rER) and extracellular matrix (ECM), and without junctions, were recognized in the interior of the EBs by transmission electron microscopy (TEM) in addition to epithelial cells. When they were stimulated by the staged-additional cocktail, they expressed albumin-positive immunoreactivity, indocyanine green (ICG) uptake, and typical ultrastructures of the hepatocytes, including bile canaliculi. These results indicate that these combined methods promote EB formation and hepatocyte differentiation from hES cells.

## INTRODUCTION

The formation of embryoid bodies (EBs) is the first important step for specific cell differentiation from embryonic stem (ES) cells[1]. In this paper, we applied an extremely simple method in order to improve the suspension culture without requiring enzymes for cell separation, which we have developed in mouse ES cells[2], to human EB formation. Next, we used these EBs for hepatocyte differentiation in order to confirm that they differentiate into a specific cell lineage. Although a few hepatocytes differentiate from ES cells only in EB medium[3], many methods that use growth factors have been developed for their effective differentiation[4,5,6,7,8,9,10]. They do not, however, always mimic the four embryonic stages of hepatic differentiation (the first, cardiac mesoderm stage; the second, septum transvesum stage; the third, hematopoietic stage; and the fourth, maturation stage)[11]; the effect of the septum transversum was especially disregarded. Previous methods were modified in this paper as a new staged-induction system by the addition of BMP-4 (bone morphogenetic protein 4) and Lanford medium, i.e., serum-free medium for maintaining human hepatocytes in culture[12].

## MATERIALS AND METHODS

KhES cell lines 1 and 3 (Institute for Frontier Medical Science, Kyoto University, Japan)[13] were used between passages 20 and 50, and maintained in 3% $CO_2$ at 37°C.

ES cell colonies on days 4 or 7 were used for EB formation. ES cell colonies, after exchange with the EB medium, DMEM/F12 (Nissui, Ibaraki, Japan) containing 20% fetal bovine serum (FBS; Hyclone, Logan, UT), were scraped in parallel three times and along the periphery directly (Cell Lifter 20CM; TRP, Switzerland) without enzymes (Fig. 1). With this operation, the colonies were scraped completely and for a short time as part of the cell sheet. Cell damage by the cell scraper was estimated by trypan blue stain. The scraped colonies were transferred into 6-cm MPC (2-methacryloyloxyethyl phosphorylcholine) plates (Lipidure®-coat; NOF, Tsukuba, Japan)[14] without pipetting, and cultured for 4 or 7 days. The medium was exchanged on day 4. It is described herein as 3SC (the Simple Static Suspension Culture method). As a control, the colony was enzymatically separated by type IV collagenase (Gibco®, Invitrogen) and trypsin (Gibco), according to the method established by Suemori et al.[13], and rotated for 4 or 7 days (RSC: Rotatory Suspension Culture).

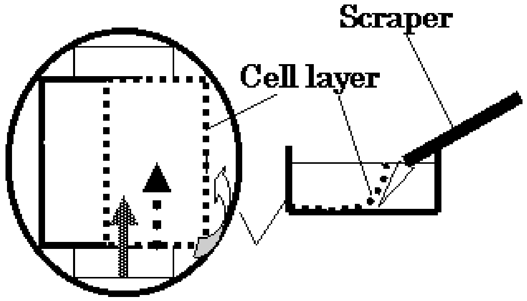

**FIGURE 1.** Cell-separation technique using a scraper. Arrows show directions of scraping.

Structural changes during EB formation were observed using an Olympus IX70 light microscope equipped with a phase-contrast apparatus (Olympus, Tokyo, Japan).

The gene expression of the ES cells of each line (days 4 and 7) and the EBs formed by 3SC and RSC were confirmed by reverse transcriptase-polymerase chain reaction (RT-PCR) (three germ-layer markers:

Nestin, Brachyury, Foxa2 primers shown in Table 1). Total RNA was extracted using TRIzol® reagent (Invitrogen). DNase-treated total RNA was used to prepare the first-strand cDNA with SuperScript® II (Invitrogen), following the protocol of the manufacturer. cDNA samples were subjected to PCR amplification with specific primers under linear conditions in order to approximate the original amount of the specific transcript. Amplification conditions included denaturation at 94°C for 5 min, followed by 30 cycles of denaturation at 94°C for 1 min, 30-sec annealing at the temperature specified for each primer, and elongation at 72°C for 30 sec. The PCR products were size-fractionated by 1.5% agarose gel electrophoresis and visualized by ethidium bromide staining. Human lip fibroblasts (JCRB91403 KD, HSRRB, Osaka, Japan) were used as the negative control.

**TABLE 1**
**Primer Sequence Information for RT-PCR**

| Gene | Primer Sequence (5′ → 3′) | Product Size (bp) | Annealing Temperature (℃) |
|---|---|---|---|
| Oct4 | Forward: CTTGCTGCAGAAGTGGGTGGAGGAA | 169 | 61 |
| | Reverse :CTGCAGTGTCGGTTTCGGGCA | | |
| Nestin | Forward: CAGCTGGCGCACCTCAAGATG | 208 | 55 |
| | Reverse: AGGGAAGTTGGGCTCAGGACTGG | | |
| Foxa2 | Forward: CTACGCCAACATGAACTCCA | 207 | 61 |
| | Reverse: AAGGGGAAGAGGTCCATGAT | | |
| Brachyury | Forward :GACAGGTACCCAACCCTGAGGA | 104 | 64.5 |
| | Reverse: AGCATGGTAAACATGCAGGTGAG | | |

Each day, the EBs were rinsed once with phosphate buffered saline (PBS) and prefixed with 2.5% glutaraldehyde in cacodylate buffer at room temperature. After overnight fixation at 4°C, they were washed several times with cacodylate buffer, postfixed with 1% $OsO_4$ for 1 h, dehydrated in an ethanol series, and embedded in an Epon-Araldite mixture after replacement with acetone. Subsequently, they were prepared for transmission electron microscopy (TEM) by a conventional method. Semi- and ultrathin sections were cut on an ultramicrotome (LKB Bromma, Stockholm, Sweden). The semithin sections were stained with toluidine blue and observed with a light microscope; the ultrathin sections were stained with uranyl acetate and lead citrate, and examined with an electron microscope (accelerating voltage: 80 kV; JEM 1200EX, JEOL, Tokyo).

EBs prepared by this method were seeded on gelatin-coated plates and differentiated by the cocktail that included 25 ng/ml acidic fibroblast growth factor (aFGF; R&D Systems, Minneapolis, MN) and 25 ng/ml basic fibroblast growth factor (bFGF; R&D Systems) for the first 3 days (the cardiac mesoderm stage), 20 ng/ml BMP-4 (R&D Systems) and 20 ng/ml hepatocyte growth factor (HGF; Pepro Tech EC, London) for the next 3 days (the septum transversum stage), and 20 ng/ml oncostatin M (OSM; R&D Systems) and 20 ng/ml vascular endothelial growth factor (VEGF; Chemicon International, Temecula, CA) for the third set of 3 days (the hematopoietic stage). After 9 days, the modified Lanford medium (Nissui Phramaceutical, Ibaraki, Japan) was used to culture the EBs for an additional 2 weeks (the maturation stage).

For immunohistochemistry, after discarding the medium, the 23-day EB outgrowth was immersed in 1% trypsin-EDTA (ethylenediaminetetraacetatic acid; Sigma, St. Louis) for 5 min at 37°C, pipetted into the medium several times, transferred onto an MPC plate, and cultured for a few days. The spheroids that formed were replated onto a gelatin-coated plate. After complete adhesion, they were washed three times with 0.02% PBS (pH 7.4) and fixed with 8% paraformaldehyde (PFA) solution (pH 7.4) for 12 h at 4°C.

They were washed three times with PBS, immersed in 0.2% Triton/PBS for 10 min, washed three times with PBS again, and then blocked with 1.5% goat normal serum/PBS (blocking solution). After washing, they were immunoreacted against a monoclonal antihuman serum albumin primary antibody (diluted to 1:1000 with blocking solution; Sigma) for 12 h at 37°C. After washing an additional three times, they were immunostained with goat antimouse Alexa 488 secondary antibody (diluted to 1:1000 with blocking solution) for 2 h and observed with a fluorescent microscope (Axio Observer.Z1:Carl Zeiss Microimaging, Germany). As control, a primary antibody was deleted.

ICG dye (25 mg; Daiichi Pharmaceutical, Tokyo), which is selectively absorbed and eliminated by hepatocytes and was previously applied to detect the differentiation of hepatocytes from mouse ES cells[9], was dissolved in 10 ml of sterile distilled water, and 2.5 ml was added to 10 ml of the modified Lanford medium (Nissui). The 23rd-day EB outgrowth stimulated by the cocktail was cultured in this solution for 1 h. After the medium was exchanged twice, the stained cells were examined with a phase-contrast microscope. A part of the specimen was prepared for TEM according to the above description.

## RESULTS AND CONCLUSIONS

The ES colony layer was separated by the scraper for a short time (usually 1 min) and only a few dead cells were detected at the edge of the peripheral zone of the colony. A large number of ES colony plaques were seen floating once the ES colony layer was transferred to MPC-coated plates. A plaque was almost identical to a single ES colony; this tendency was the same for KhES cell lines 1 and 3. The processes of EB formation were as follows: aggregates of large cells appeared in the plaque after 24 h; they were often found among a bush consisting of feeder cells. The surface of the primary EB was undulated; there were subtle differences between the cell lines. KhES 3 formed large, relatively round EBs, whereas KhES 1 had small, poorly formed EBs. Approximately on day 3, the cellular aggregates compacted and formed completed structures, like the morula, which did not fuse with each other after this stage. Subsequently, the cellular aggregates formed one or more central or peripheral coeloms, such as blastocysts (Fig. 2a,b) that gradually enlarged, became relatively transparent, and slightly protruded in the periphery.

The occurrence of three germ layers in EBs formed by 3SC and RSC was confirmed (Fig. 2c). Brachyury was weak, except in cell line 1; day 7; 3SC after 30 cycles of RT-PCR in EBs of either of the cell lines cultured by either of the methods, although it could be confirmed in each lane. Nestin and Foxa2, ectodermal and endodermal markers, respectively, occurred in all lines, methods, and conditions except in cell line 1; day 4; RSC; which showed weak reactions to all markers. In the negative control of human lip fibroblast, these genes were not detected.

Each EB on day 7 contained mesenchymal cells with well-developed rough endoplasmic reticulum (rER), a rich extracellular matrix (ECM), and collagenous fibers in their interior, even though the columnar epithelium developed on its surface (Fig. 2d). Although these processes were like those seen in mice[2], the appearance of mesenchymal cells was more rapid and definite in hES cells.

After then, this experiment focused on hepatocyte differentiation in order to prove that EBs formed by the method can differentiate hES cells into specific cells. Our strategy of differentiation was to mimic embryonic stages. The importance of BMP-4, which plays a chief role in the septum transversum stage, in hepatocyte differentiation from mouse ES cells was first pointed out by Gouon-Evans et al.[15], although they did not imitate embryonic stages in their experimental design. Although analysis by RT-PCR of a hepatocyte marker gene in another cell line (H1)[16] suggested the appearance of Hhex, G-6P, TAT, albumin in the second stage, i.e., BMP-addition stage (data not shown here), the effects of BMP-4 were not so definite in morphology; at least, hepatocyte-like cells were not confirmed by the phase contrast microscopy. However, the modified Lanford medium promoted the differentiation of ES cells into albumin-producing cells easily and powerfully, despite the fact that it was primarily developed in order to maintain the long-term primary culture of human hepatocytes[12] and used in this study only for maturation. First, hepatocyte-like cells with two nuclei appeared about day 14 after immersion in the Lanford medium (Fig. 3a), which absorbed ICG (Fig. 3b) and were immunostained with albumin (Fig. 3c). TEM images showed

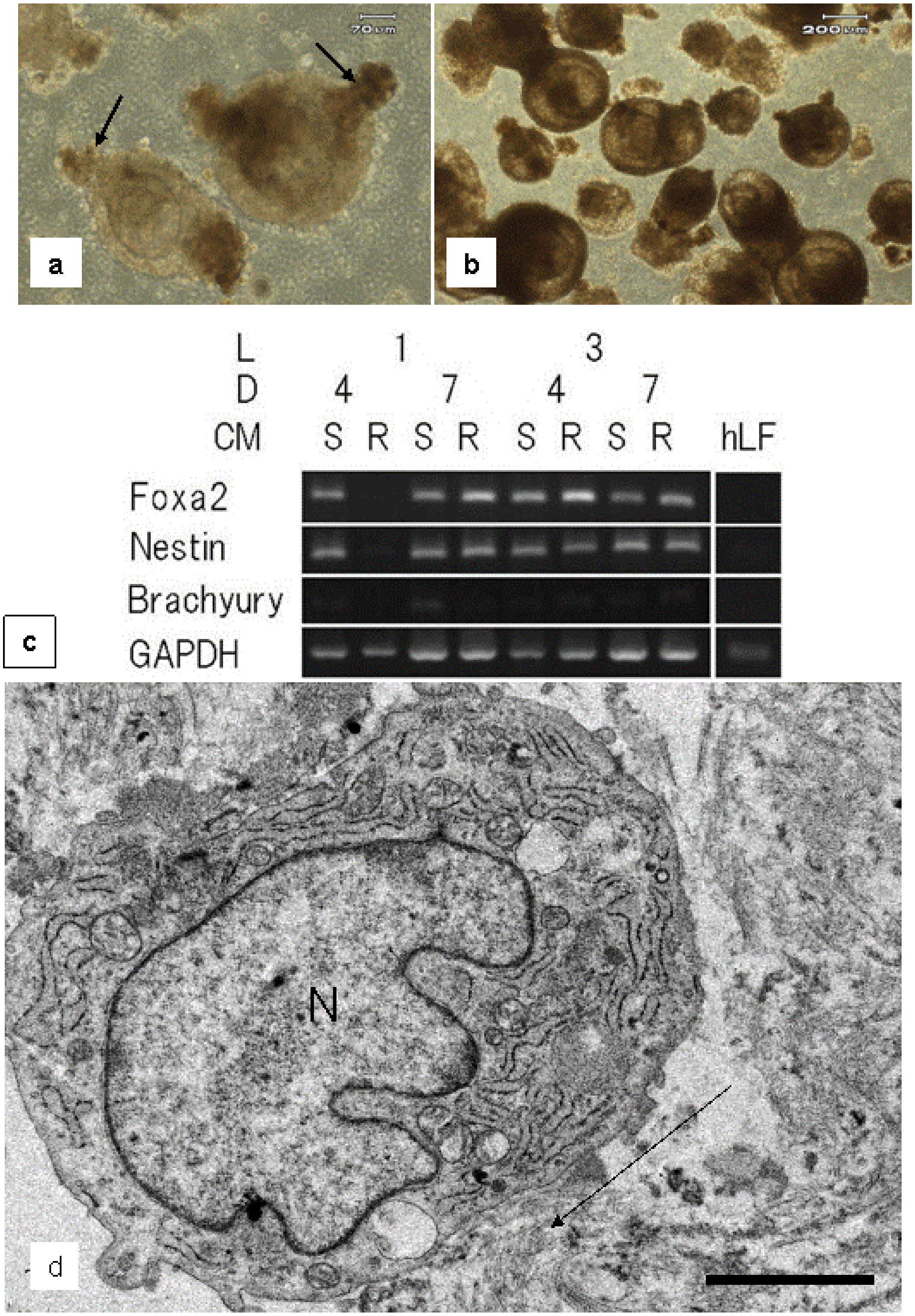

**FIGURE 2.** (a) EB formation on day 7 (KhES 1). Each EB has a central coelom. Small cell condensations formed on the periphery like buds (arrows). (b) EB on day 7 (KhES 3). Many EBs contained double coeloms. (c) Three germ-layer markers are detected in all EBs, although brachyury is weak under the same conditions as the other markers. L: cell line, D: culturing days, CM: culture method, S: 3SC, R: RSC, hLF: control. (d) Ultrastructural features of the mesodermal-originating cells in a KhES 1–derived EB on day 7. A round cell contains rich rER and ECM with microfibrils (arrow) and does not conjugate with other cells. N: nucleus. Bar = 2 µm.

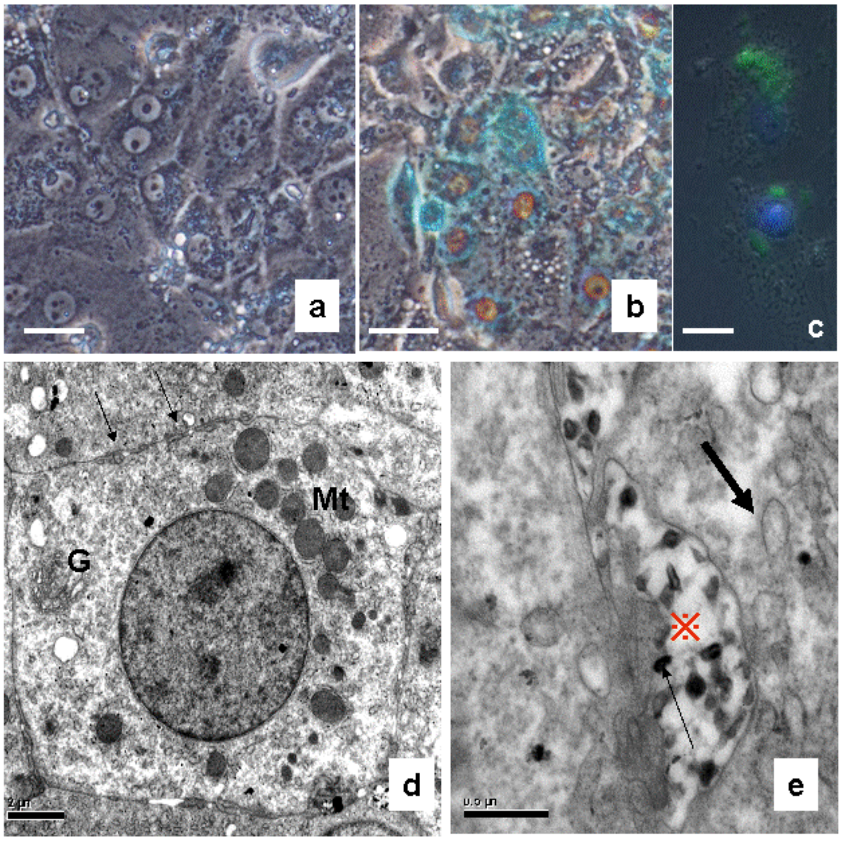

**FIGURE 3**. Hepatocyte differentiation (KhES 3). (a) Phase-contrast image of EB outgrowth on day 23. The cell cluster consists of large cells with a round nucleus or two nuclei. Bar = 70 µm. (b) Partial uptake of ICG (green) in the cell cluster. Bar = 70 µm. (c) Green-fluorescent albumin-positive large cells contain many intracellular organellae. Bar = 20 µm. (d) TEM image of the ICG-positive cell that contains a large round nucleus, rich round mitochondria (Mt), Golgi apparatus (G), and bile canaliculi (arrows). Bar = 2 µm. (e) Bile canaliculi (shown in red) develop well in these cells, in which high-density structures are plentiful and seem to be associated with ICG granules (fine arrow). Irregular vesicles without ribosomes are distributed near the canaliculus, suggesting poorly developed sER (bold arrow). Bar = 0.5 µm.

typical hepatocytes with large round nuclei (Fig. 3d), developed rER, and well-recognized bile canaliculi (Fig. 3e). These results indicate that EBs formed by this simple method are differentiated into a specific cell lineage and the developed cocktail, especially the modified Lanford medium, is effective for hepatocyte differentiation.

## ACKNOWLEDGMENTS

We thank Dr. Kametani K and Ms. Suzuki K (Research Center for Instrumental Analysis of Shinshu University) for their excellent technical assistance.
